# Supplementary material for: Identification of photoperiod-induced specific miRNAs in the adrenal glands of Sunite sheep (Ovis aries)
Source: Front Vet Sci. 2022 Jul 22;9:888207. doi: 10.3389/fvets.2022.888207 (PMC9354845; doi:10.3389/fvets.2022.888207)
Supplement: Supplementary file 1 [file Data_Sheet_1.ZIP › Supplementary file/miRDeep2 file.zip!/miRDeep2 file/Know_Structure/SPLP42A3/oar-mir-27a.pdf]

miRBase precursor : oar-mir-27a
Total read count : 136427
oar-miR-27a read count : 136034
remaining reads : 393

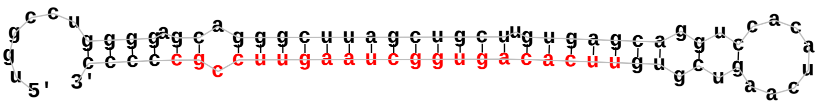

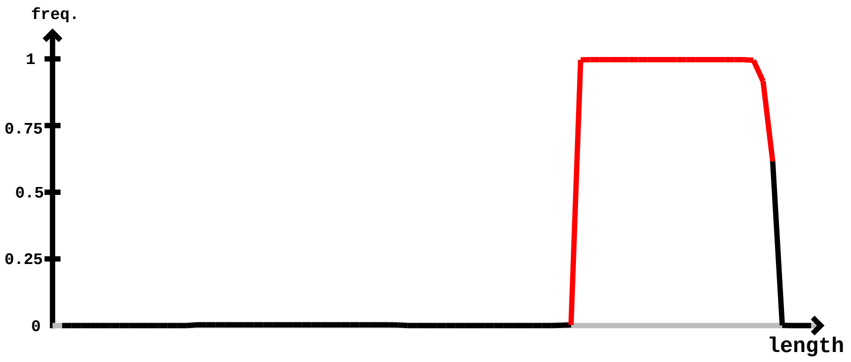

oar-miR-27a

```
5'- uggccuggggagcagggcuuagcugcuugugagcagguccacaucaagucguguucacaguggcuaaguuccgccccc   -3'    exp
    ......((((.((.(((((((((((((.(((((((.(.(........).).)))))))))))))))))))).))))))   reads   mm   sample
    .............agggcuuagcugcuugug...............................................   1       0    seq
    .............agggcuuagcugcuugugag.............................................   1       0    seq
    .............agggcuuagcugcuugugagc............................................   76      0    seq
    .............agggcuuagcugcuugugagca...........................................   301     0    seq
    .............agggcuuagcugcuugugagcagg.........................................   7       0    seq
    .............agggcuuagcugcuugugagcaggu........................................   5       0    seq
    .............agggcuuagcugcuugugagcagguccacaucaagucgug.........................   1       0    seq
    ..............gggcuuagcugcuugugagca...........................................   1       0    seq
    ...................................................uguucacaguggcuaaguuc.......   1       0    seq
    ...................................................uguucacaguggcuaaguucU......   5       1    seq
    ...................................................uguucacaguggcuaaguucc......   11      0    seq
    ...................................................uguucacaguggcuaaguuccg.....   116     0    seq
    ...................................................uguucacaguggcuaaguuccgc....   20      0    seq
    ...................................................uguucacaguggcuaaguuccgcc...   3       0    seq
    ....................................................guucacaguggcuaaguucU......   9       1    seq
    ....................................................guucacaguggcuaaguucc......   3       0    seq
    ....................................................Auucacaguggcuaaguucc......   1       1    seq
    ....................................................guucacaguggcuaaguucUg.....   26      1    seq
    ....................................................guucacaguggcuaaguuccgc....   1       0    seq
    ....................................................guucacaguggcuaaguucUgc....   158     1    seq
    .....................................................uucacaguggcuaaguu........   21      0    seq
    .....................................................Aucacaguggcuaaguu........   2       1    seq
    .....................................................uucacaguAgcuaaguuc.......   2       1    seq
    .....................................................uucacaguggcuaaguuc.......   312     0    seq
    .....................................................uucacaguggcuaaguucU......   8769    1    seq
    .....................................................uucacaguggcuaaguucc......   1875    0    seq
    .....................................................uucacaguggcuaaguucUg.....   8288    1    seq
    .....................................................uucacaguggcuaaguuccg.....   26960   0    seq
    .....................................................uucacaguggcuaaguuccA.....   5564    1    seq
    .....................................................uucacaguggcuaaguucUgc....   79533   1    seq
    .....................................................uucacaguggcuaaguuccgc....   4123    0    seq
    .....................................................uucacaguggcuaaguuccgcc...   107     0    seq
    ......................................................ucacaguggcuaaguuc.......   1       0    seq
    ......................................................ucacaguggcuaaguucc......   2       0    seq
    ......................................................ucacaguggcuaaguucU......   7       1    seq
```

oar-miR-27a

```
uggccuggggagcagggcuuagcugcuugugagcagguccacaucaagucguguucacaguggcuaaguuccgccccc
......................................................ucacaguggcuaaguuccg.....    23    0    seq
......................................................ucacaguggcuaaguuccA.....    3     1    seq
......................................................ucacaguggcuaaguucUg.....    7     1    seq
......................................................ucacaguggcuaaguucUgc....    63    1    seq
......................................................ucacaguggcuaaguuccgc....    9     0    seq
.......................................................cacaguggcuaaguucc......    1     0    seq
.......................................................cacaguggcuaaguucU......    2     1    seq
.......................................................cacaguggcuaaguuccg.....    1     0    seq
.......................................................cacaguggcuaaguucUg.....    1     1    seq
.......................................................cacaguggcuaaguucUgc....    3     1    seq
........................................................acaguggcuaaguuccg.....    1     0    seq
```
